# A novel mechanism of cell growth regulation by Cell Cycle and Apoptosis Regulatory Protein (CARP)-1

**DOI:** 10.1186/1750-2187-5-7

**Published:** 2010-07-01

**Authors:** Yan Jiang, Vineshkumar T Puliyappadamba, Liyue Zhang, Wenjuan Wu, Anil Wali, Michael B Yaffe, Joseph A Fontana, Arun K Rishi

**Affiliations:** 1 Department of Internal Medicine, Wayne State University and John D. Dingell VA Medical Center, Room B4325, 4646 John R, Detroit, MI 48201, USA; 2 Karmanos Cancer Institute, Wayne State University and John D. Dingell VA Medical Center, Room B4325, 4646 John R, Detroit, MI 48201, USA; 3 Department of Surgery, Wayne State University, and John D. Dingell VA Medical Center, Room B4245, 4646 John R, Detroit, MI 48201, USA; 4 Biology Department, Massachusetts Institute of Technology, Cambridge, MA 02115, UK; 5 Department of Biochemistry, University of Western Ontario Schulich School of Medicine and Dentistry, 4th Floor Victoria Research Labs, A4-130a 800 Commissioners Road East, London, ON N6C 2V5, UK; 6 Department of Obstetrics & Gynecology, University of Western Ontario Schulich School of Medicine and Dentistry, 4th Floor Victoria Research Labs, A4-130a 800 Commissioners Road East, London, ON N6C 2V5, UK

## Abstract

**Background:**

CARP-1/CCAR1, a perinuclear phospho-protein, regulates signaling by adriamycin, steroids, or growth factors. However, intracellular events that regulate CARP-1-dependent cell growth are not fully understood.

**Results:**

Here we investigated whether CARP-1 is involved in signaling induced by the protein kinase A inhibitor H89. Treatments of human breast cancer cells with H89 resulted in apoptosis that involved enhanced CARP-1 threonine phosphorylation and expression. Depletion of CARP-1, on the other hand, abrogates apoptosis induced by H89. CARP-1 binds with signal transducer TAZ and over-expression of TAZ inhibits apoptosis by CARP-1. CARP-1 (651-759) interacts with a novel, N-terminal epitope of TAZ. H89 treatment stimulates threonine phosphorylation of CARP-1 (651-759), while substitution of threonine^667 ^to alanine interferes with its binding with TAZ and apoptosis by H89. In addition, expression of wild type or CARP-1 (651-759) causes loss of c-myc expression due, in part, to suppression of c-myc transcription.

**Conclusions:**

CARP-1 threonine^667 ^regulates H89-dependent signaling by a novel pathway that involves modulation of CARP-1 interaction with TAZ and transcriptional down-regulation of c-myc.

## Background

Apoptosis is essential in maintaining tissue homeostasis in a host of conditions including development, wound healing, and elimination of infectious pathogens. Defective apoptosis is often encountered in many diseases including cancer [1,2]. Although, anticancer therapeutics function in part by targeting apoptosis pathways, development of drug resistance remains a problem and therefore warrants identification and exploitation of additional apoptosis transducers to effectively manage drug-resistant cancers. CARP-1/CCAR1 is a perinuclear protein that functions in regulating signaling by growth factors as well as chemotherapeutics such as adriamycin, etoposide, and iressa [3,4]. CARP-1 is a phospho-protein that is a target of phosphorylation by the DNA-damage induced ATM kinase [5], and serves as a key co-activator of the steroid/thyroid receptor family of transcription factors as well as tumor suppressor p53 [6]. Although ectopic expression of CARP-1 diminishes levels of cell-cycle regulatory proteins such as c-myc, cyclin B, and topoisomerase IIα [3,4], the mechanisms by which CARP-1 regulates apoptosis and its role in various pathways that regulate cell growth are yet to be fully elucidated.

We previously found that CARP-1 binds with 14-3-3/stratifin [3]. 14-3-3 proteins belong to a family of highly conserved and ubiquitously expressed proteins that regulate differentiation, cell cycle progression and apoptosis signaling by binding with diverse intracellular proteins in a manner dependent or independent of their phosphorylation [7]. TAZ, a transcriptional co-activator that has a conserved WW, a coil-coil, a transactivation domains, as well as has a C-terminal PDZ binding motif, is a ligand for 14-3-3 proteins [8]. TAZ is a negative regulator of peroxisome proliferator-activated receptor γ-dependent transcription, functions as a modulator of mesenchymal stem cell differentiation by promoting Runx-2-dependent transcription, and is involved in development of multiple organs [9]. Lats kinase phosphorylates TAZ at serine 89 that in turn promotes its 14-3-3-mediated nuclear export with consequent inhibition of its transcriptional co-activation function [8,10]. Since, CARP-1 is also a ligand of 14-3-3, the extent CARP-1 regulates signaling involving TAZ is unclear.

Signaling by PKA has been implicated in numerous cellular processes that include modulation of other protein kinases, regulation of intracellular calcium and transcription [11]. H89, a compound characterized in vitro as a potent and selective inhibitor of PKA, is a competitive antagonist of ATP at its binding site on the PKA catalytic subunit and therefore has been extensively used to study PKA functions [12,13]. A number of recent studies however have identified actions of H89 that are independent of its effects on PKA [reviewed in [14]], suggesting likely involvement of multiple pathways in transducing intracellular signaling by this compound. In this context, a recent report revealed involvement of a nuclear hormone receptor co-activator, NRIF3, in regulating H89-dependent apoptosis in breast cancer cells [15]. Since CARP-1 also associates with components of the mediator complex to regulate expression of ER and GR target genes, as well as functions as a p53 co-activator to transduce apoptosis by chemotherapeutic adriamycin [6], we investigated whether CARP-1 was also involved in regulating cell growth inhibitory signaling by H89. Apoptosis signaling induced by H89 caused elevated threonine phosphorylation as well as expression of CARP-1, while depletion of CARP-1 interfered with H89 effects. H89 regulation of CARP-1 interaction with TAZ and consequent repression of c-myc elaborate a novel mechanism of cell growth inhibition.

## Results

### H89 induces apoptosis, CARP-1 expression and threonine phosphorylation, while loss of CARP-1 prevents apoptosis by H89

**Figure 1:**
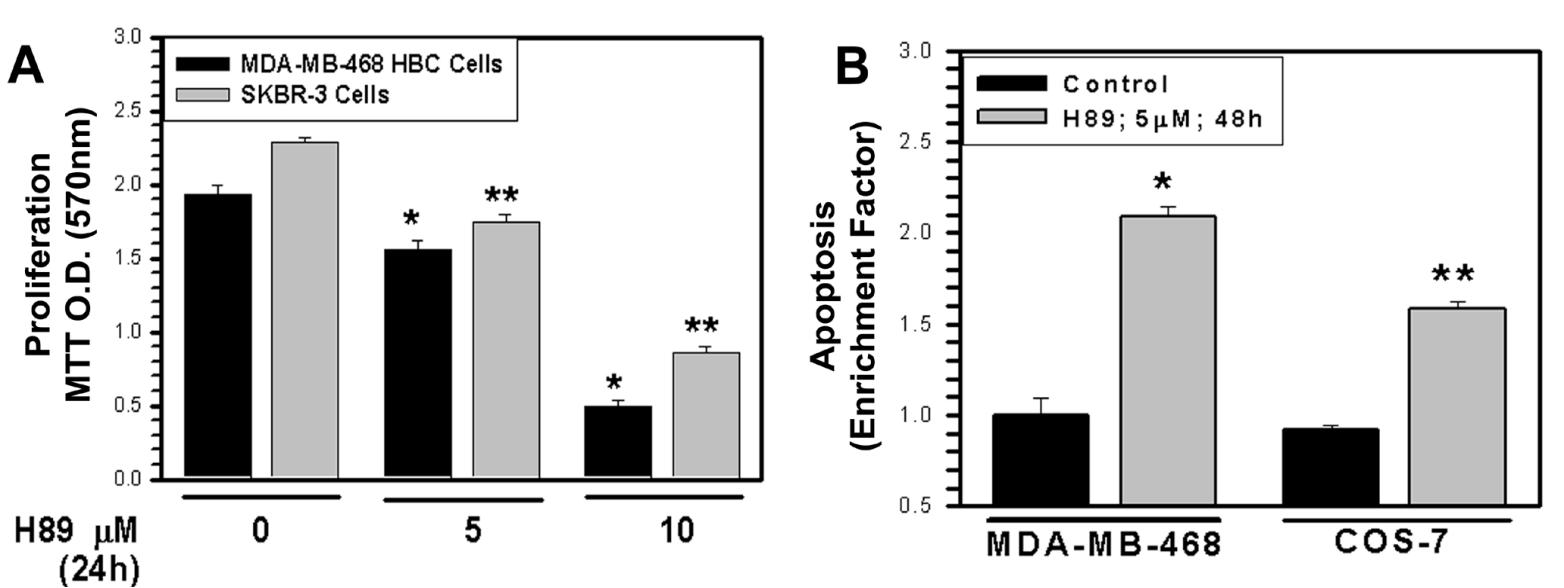
**H89 attenuates cell proliferation (A) and enhances apoptosis (B)**. Cells growing in normal serum conditions were either untreated (noted as 0) or treated with noted doses of H89 for indicated time, followed by determination of cell proliferation by MTT assay (panel A) or apoptosis (panel B) as in methods. Columns in histograms in panels A and B represent data from 3 independent experiments; bars, SE. Data in both panels were analyzed using a two-tailed Student *t *test. For panel A, *P and **P < 0.0018 and 0.00002, respectively, and for panel B, *P and **P < 0.000026 and 0.0003, respectively, compared to the corresponding untreated controls.

To test whether H89 treatments induce apoptosis and the extent CARP-1 is involved in growth inhibitory signaling by H89, we first determined growth inhibition of HBC and COS-7 cells by this agent. Exposure to H89 inhibited cell growth (Figure 1A), in part, by enhancing apoptosis (Figure 1B). Since H89 functions as an antagonist of the PKA catalytic subunit (PKA-C), as well as inhibits MSK1 and ribosomal S6Kinase [14], we next determined whether H89 signaling targeted these kinases to elicit cell growth inhibition. For this purpose, we knocked-down each of these kinases by utilizing on-target-plus Si-RNAs. Western blot analyses of the HBC cells transfected separately with respective Si-RNAs revealed a greater than 70% to almost complete loss of PKA-Cα, S6K, or MSK1 expression when compared with corresponding scrambled Si-RNA-transfected controls, and the targeted depletion of either of these kinases failed to induce apoptosis (not shown). These preliminary findings raise the possibility that different perhaps novel signaling pathway(s) are targeted by H89 to inhibit cell growth.

**Figure 2:**
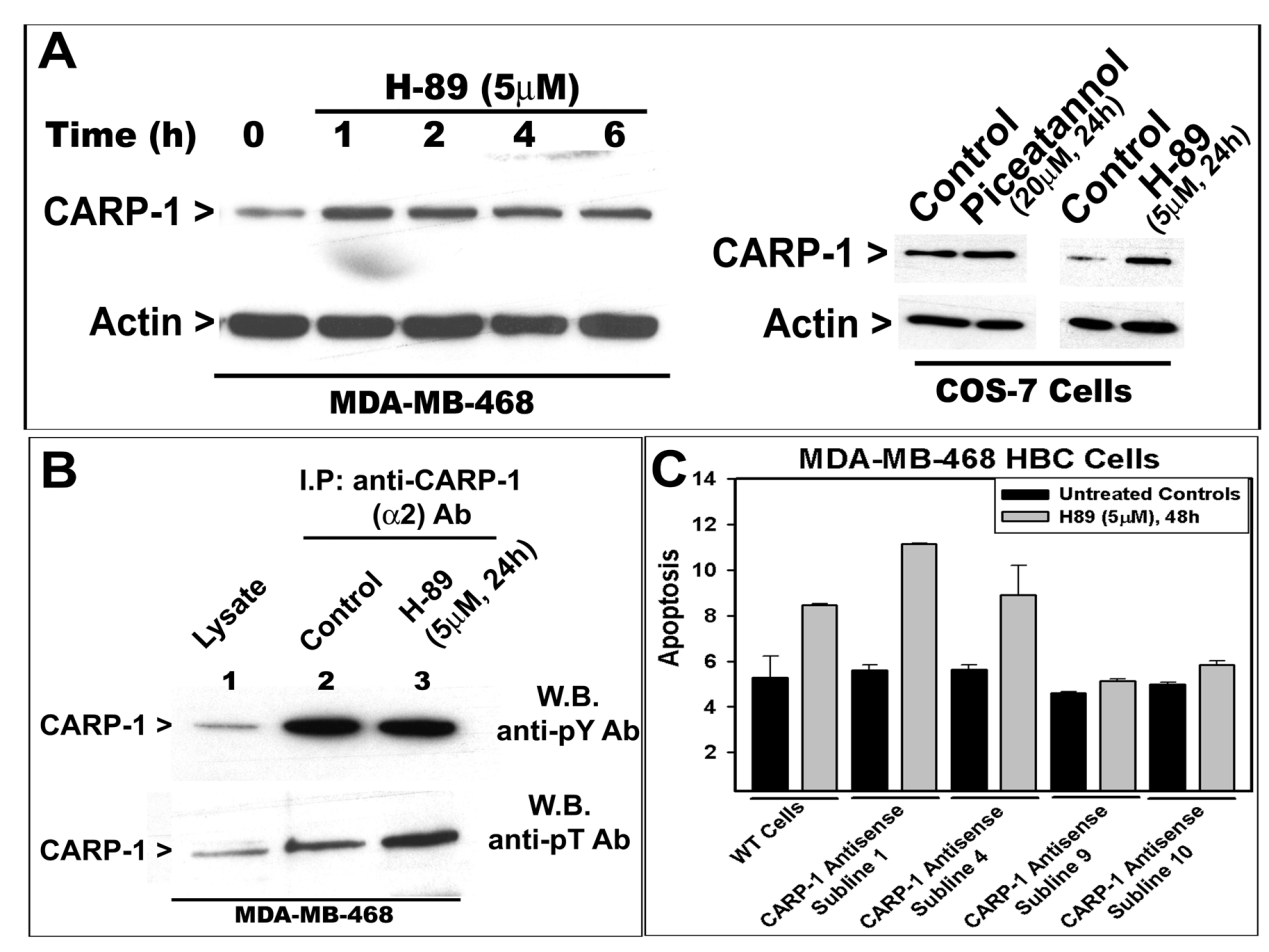
**H89 induces CARP-1 expression (A) and threonine phosphorylation (B), and depletion of CARP-1 abrogates apoptosis by H89 (C)**. In panel A, the cells were treated with noted dose of H89 or piceatannol for indicated time periods. Approximately 100 μg of respective protein lysates were electrophoresed on 10% SDS-PAGE, followed by western immunoblotting. The membranes were first probed with anti-CARP-1 (α2) antibody [3], followed by probing with anti-actin antibody to assess loading. In panel B, cells were either untreated (Control) or treated with H89 for indicated dose and time, and cell lysates were prepared. In lanes 2 and 3, co-immunoprecipitation using noted antibodies and 1000 μg protein lysate was carried out prior to western blotting. In lane 1, 100 μg protein lysate was analyzed along with immunoprecipitates on 10% SDS-PAGE followed by immunoblotting with anti-phospho-tyrosine or anti-phospho-threonine antibodies. In panel C, HBC cells expressing normal (indicated as CARP-1 antisense sublines 1, 4) or 50% reduced CARP-1 (indicated as sublines 9, 10) were either untreated or treated with noted time and dose of H89, and apoptosis determined as in figure 1D. Columns in histogram represent data from 3 independent experiments; bars, SE.

Whether apoptosis signaling by H89 involves CARP-1 was investigated next. CARP-1 levels were elevated in cells that were treated with H89 or piceatannol (Figure 2A). Studies from our laboratory as well as by others have revealed that CARP-1 is a phosphoprotein [4,16]. We further investigated whether H89 signaling targeted CARP-1 phosphorylation. HBC cells were either untreated or treated with H89, and the CARP-1 protein was immunoprecipitated with anti-CARP-1 (α2) polyclonal antibodies [3], followed by western blot analysis with anti-phospho-tyrosine or anti-phospho-threonine antibodies. The data revealed that while H89 did not cause elevated tyrosine-phosphorylation of CARP-1, the level of threonine phosphorylated CARP-1 was elevated following exposure to H89 (Figure 2B). Thus, H89 treatments cause increased CARP-1 expression as well as its threonine phosphorylation.

The role of CARP-1 in regulating H89-dependent apoptosis was further examined by utilizing multiple MDA-MB-468 HBC sublines that stably express CARP-1 antisense as in methods. Wild-type HBC cells or CARP-1 antisense transfected sublines 1, 4, 9, and 10 were either untreated or treated with H89, and cell lysates analyzed for apoptosis as in methods. As shown in Figure 2C, H89-dependent apoptosis was inhibited in cells that had reduced levels of CARP-1 (sublines 9, 10) when compared to those with normal levels of CARP-1 (wild-type, and sublines 1, 4). Taken together, these results demonstrate that H89-dependent inhibition of HBC cell growth is likely independent of its targeting the PKA, S6K, or MSK1 kinases, while involves elevated threonine phosphorylation and expression of CARP-1. Moreover, since depletion of CARP-1 interferes with apoptosis by H89, our data further underscore an important role for CARP-1 in signaling by H89.

### CARP-1 interacts with signal transducer TAZ, and expression of TAZ inhibits CARP-1-dependent apoptosis

**Figure 3:**
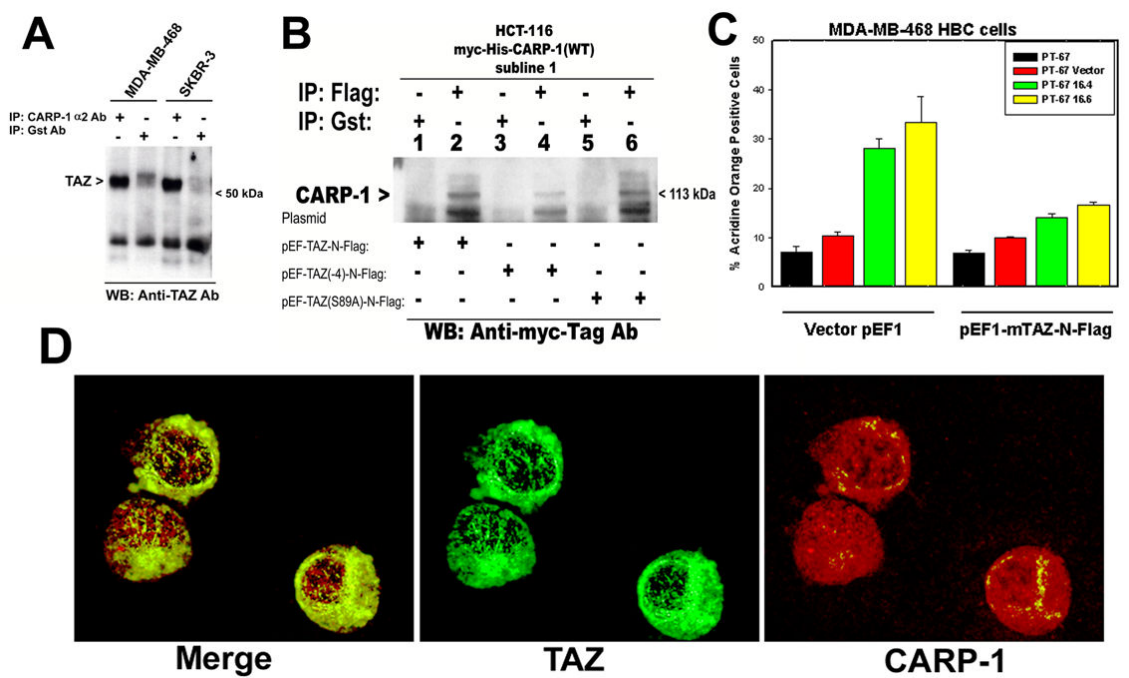
**CARP-1 interacts with TAZ (A, B), TAZ expression interferes with CARP-1-dependent apoptosis (C), and CARP-1 and TAZ co-localize in cytoplasmic/perinuclear region (D)**. For CARP-1 binding with TAZ, Breast cancer cells (panel A) or HCT-116 colon cancer cells expressing myc-His-tagged wild-type CARP-1 (panel B) were utilized. In panel B, the cells were transfected with plasmids encoding wild-type or mutant TAZ proteins as indicated, prior to generating cell lysates. The cell lysates were then utilized for immunoprecipitation and western blotting using noted antibodies essentially as in figure 2C. In panel C, cells were transfected with plasmids encoding vector or wild-type TAZ followed by their transduction with indicated retroviruses expressing vector or wild-type CARP-1 as detailed in methods. The apoptosis levels were assessed by determining the acridine orange-positive cells as described before [4]. Columns in histogram represent data from 3 independent experiments; bars, SE. In panel D, the cells were transfected with a combination of plasmids encoding myc-His-tagged wild-type CARP-1 and flag-tagged wild-type TAZ proteins, followed by their fluorescent labeling and photography as described in methods.

We have previously noted that expression of 14-3-3/stratifin interferes with apoptosis by CARP-1, and that CARP-1 interacts with 14-3-3/stratifin [3]. Moreover, 14-3-3 was also found to bind with TAZ protein in a manner dependent on TAZ serine phosphorylation [8]. Together with the available GenBank database information indicating CARP-1 as a TAZ-binding protein, we speculated that CARP-1 may be part of a multi-protein cytoplasmic complex, and that CARP-1 interactions with 14-3-3 and/or TAZ regulate signaling for cell growth. To test this possibility, we investigated whether CARP-1 interacts with TAZ, the molecular basis of their interaction and the extent these interaction regulates apoptosis. In the first instance, the cell lysates derived from MDA-MB-468 and SKBR-3 breast cancer cells were subjected to immunoprecipitation with anti-CARP-1 (α2) polyclonal antibodies [3] followed by western immunoblotting with anti-TAZ antibodies. As shown in figure 3A, TAZ protein was detected in precipitates derived from anti-CARP-1 antibodies but not from anti-Gst-tag antibodies, suggesting specific interaction between cellular CARP-1 and TAZ proteins. We further ascertained CARP-1 interaction with TAZ by utilizing HCT-116 colon cancer cells that stably express myc-His-tagged wild-type CARP-1. These cells were transfected with plasmids encoding flag-tagged wild-type TAZ (pEF-TAZ-N-Flag) or mutant TAZ proteins (pEF-TAZ(-4)-N-Flag and pEF-TAZ(S89A)-N-Flag that encode TAZ lacking PDZ binding and serine phosphorylation sites, respectively). The TAZ proteins from respective cell lysates were immunoprecipitated with anti-Flag-tag or anti-Gst-tag antibodies, and the immunoprecipitates subjected to western blot analysis with anti-myc-tag antibody. The data revealed the presence of CARP-1 in the immunoprecipitates derived from anti-Flag-tag (lanes 2, 4, and 6) but not from anti-Gst-tag (lanes 1, 3, and 5) antibodies (Figure 3B), suggesting that CARP-1 binding with TAZ does not require the PDZ motif or serine at position 89 of TAZ. Whether TAZ plays a role in CARP-1-dependent apoptosis was investigated next. HBC cells were transfected with vector plasmid pEF1 or pEF1-TAZ-N-Flag followed by incubation of cells with retroviruses encoding vector or myc-His-tagged CARP-1 for a period of 48 h. CARP-1 transduction of cells expressing vector plasmid resulted in ~3-fold increase in apoptosis when compared with their vector transduced counterparts, whereas expression of TAZ inhibited apoptosis induced by CARP-1 (Figure 3C). Since TAZ shuttles between cytoplasmic and nuclear compartments [8] and CARP-1 is a perinuclear protein [3], for their binding to occur, we ascertained the extent the two proteins co-localize. To address this issue, the COS-7 cells were transfected with a combination of plasmids encoding Flag-tagged wild-type TAZ and myc-His-tagged wild-type CARP-1, followed by immunocytochemical detection of fluorescently-labelled respective tagged proteins. As shown in Figure 3D, both the proteins localized predominantly in the cytoplasmic compartment with punctuate nuclear presence.

**Figure 4:**
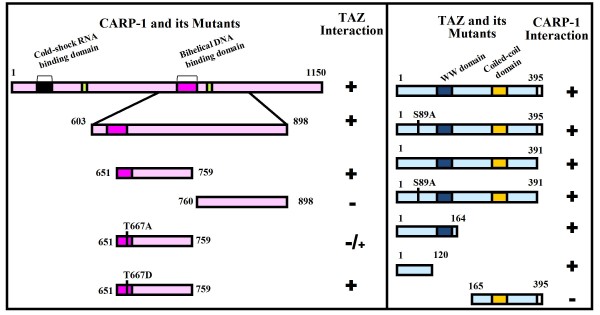
**CARP-1 (651-759) binds with TAZ (1-120)**. Schematic diagram of CARP-1 (WT and its various mutants; Left Box) and TAZ (WT and its mutants; Right Box) constructs that were utilized to map minimal epitopes for their interaction. All CARP-1 proteins have myc and 6 × His epitopes at their carboxyl termini. All the TAZ proteins, with the exception of 1-120, 1-164, and 165-395, harbored flag epitope at their amino termini, while TAZ 1-120 and its 1-164 and 165-395 mutants had Gst epitope at their amino termini. Positive interactions are indicated by + and loss/absence of interaction is denoted by -.

To map TAZ-interacting domain of CARP-1, cells were transfected with plasmid encoding flag tagged TAZ in combination with the vector plasmid or recombinant plasmids encoding various myc-His-tagged CARP-1 mutants that have been described before [4]. Cell lysates were utilized to immunoprecipitate CARP-1 proteins using anti-myc-tag antibodies followed by western blotting in conjunction with anti-flag tag antibody. The data revealed that TAZ-interacting epitope is present within CARP-1 amino acids 651-759 (Additional file 1A). Similar experimental strategy was utilized to map CARP-1-interacting epitope of TAZ by expressing myc-His-tagged CARP-1 (651-759) mutant in combination with various plasmids encoding gst-tagged TAZ proteins. We found that CARP-1 interacted with a novel epitope within the N-terminal of TAZ, and that TAZ serine 89, WW, PDZ or coiled-coil motifs were not involved in its binding with CARP-1 (Additional file 1B). Our mapping studies are summarized in figure 4, and together with the data in figures 2 and 3 demonstrate that novel motifs of CARP-1 and TAZ are involved in their binding, and that TAZ expression interefers with apoptosis signaling by CARP-1.

### CARP-1 threonine at position 667 is a target of H89-dependent apoptosis signaling

**Figure 5:**
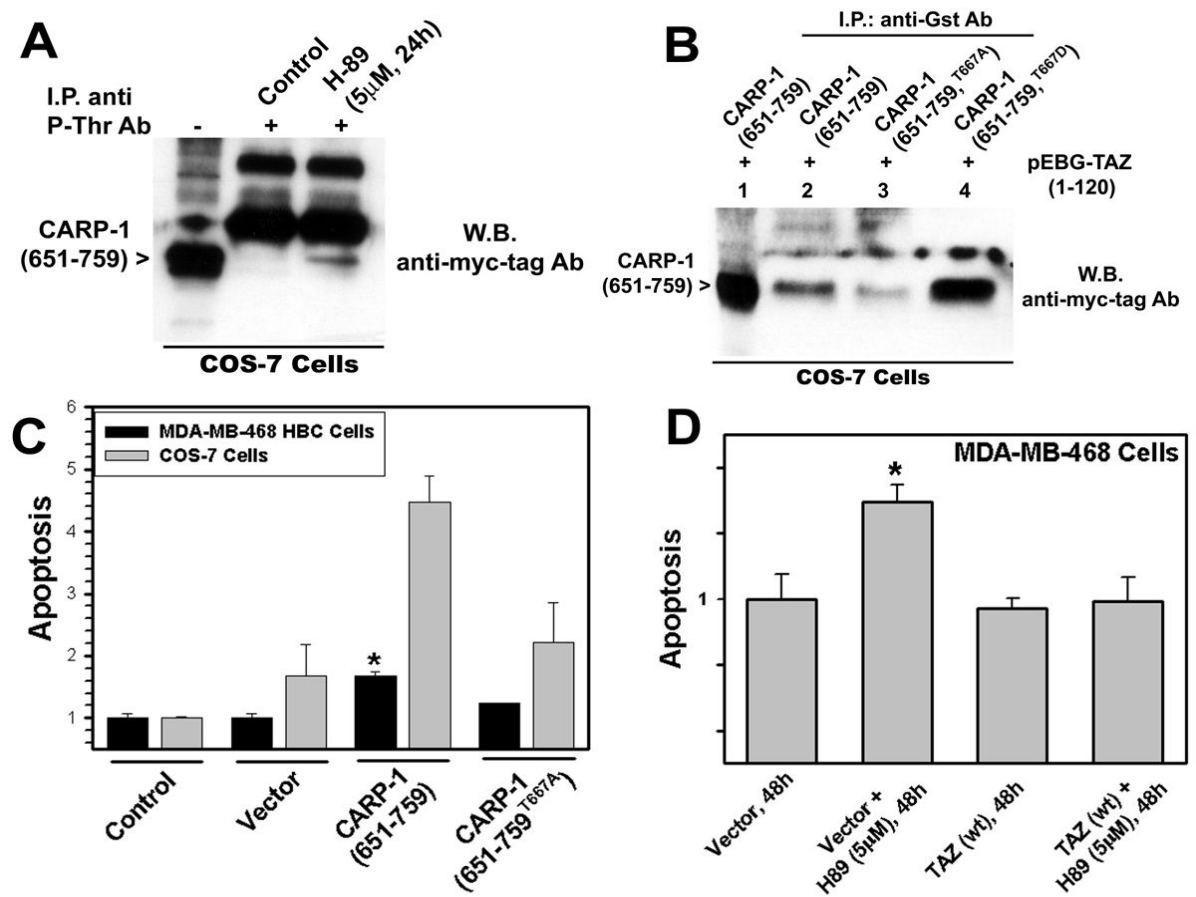
**CARP-1 threonine^667 ^is a target of H89 signaling, regulates CARP-1-TAZ binding, and CARP-1-dependent apoptosis**. (**A**) Cells transfected with plasmid encoding myc-His-tagged CARP-1 (651-759) mutant were either untreated (Control) or treated with H89 for indicated dose and time, and cell lysates were prepared, and protein lysate subjected to immunoprecipitation using phospho-theronine antibodies (noted as P-Thr Ab), and protein lysate (100 μg, lane indicated with -) along with immunoprecipitates (lanes indicated with +) analyzed by western blotting with anti-myc-tag antibody. (**B**) Cells were transfected with plasmid encoding flag-tagged TAZ (1-120) mutant [indicated as pEBG-TAZ (1-120)] in combination with plasmids expressing noted myc-His-tagged CARP-1 mutant proteins. The cell lysates in lanes 2, 3, and 4 were subjected to immunoprecipitation using anti-Gst tag antibodies essentially as in figure 4B. Protein lysate and immunoprecipitates analyzed by western blotting as in panel A. Presence of CARP-1 (651-759) proteins is indicated on the left side of each panel. (**C) **Cells were either untransduced (Control), or transduced with retroviruses expressing vector or myc-His-tagged CARP-1 mutants. (**D**) Cells were transduced with retroviruses encoding vector plasmid or wild-type TAZ followed by treatments with H89 for noted dose and time. Protein lysates in both panels C and D were assayed for apoptosis essentially as in figure 1D. Columns in histograms represent data from 2 independent experiments; bars, SE. Data in panels C and D were analyzed using a two-tailed Student *t *test. For panel C, *P < 0.005, while for panel D, *P < 0.0087, compared to the corresponding vector controls.

To define mechanisms of H89-dependent apoptosis, we first determined whether TAZ-interacting CARP-1 (651-759) mutant is a target of H89 signaling. To address this issue, cells expressing myc-His-tagged CARP-1 (651-759) protein were either untreated or treated with H89. The protein complex was immunoprecipitated with anti-phospho-threonine antibodies, followed by western blotting with anti-myc-tag antibodies. H89 induced threonine phosphorylation of CARP-1 (651-759) protein when compared with the untreated control (Figure 5A) indicating that H89 signaling targets threonine residue(s) within CARP-1 (651-759) protein. Since CARP-1 (651-759) mutant also harbors TAZ-binding epitope (figure 4), we next determined whether threonine phosphorylation of CARP-1 (651-759) regulates CARP-1 binding with TAZ. On the basis of Prosite database analysis [17] that predicted CARP-1 threonine^667 ^as a high probability phosphorylation site, we generated additional constructs for expression of CARP-1 (651-759) protein with threonine^667 ^substituted to either alanine (T667A) or aspartate (T667D) as detailed in methods. Cells expressing Gst-TAZ (1-120) in combination with myc-His-tagged CARP-1 (651-759), CARP-1 (651-759^T667A^), or CARP-1 (651-759^T667D^) proteins were utilized to analyze protein complexes that were immunoprecipitated with anti-Gst-tag antibodies followed by western blotting with anti-myc-tag antibodies. The data revealed that TAZ binding with CARP-1 (651-759^T667A^) protein was attenuated (Figure 5B, lane 3). T667D substitution in CARP-1 (651-759) protein, on the other hand, enhanced its binding with TAZ (Figure 5B, lane 4) when compared with binding of CARP-1 (651-759) with TAZ. Since substitution of phospho-threonine or phospho-serine residue(s) to aspartate often mimics phosphorylated state of a protein, it is likely that threonine^667 ^to aspartate change allows a threonine phosphorylated conformation of CARP-1 (651-759), and favors its binding with TAZ.

Whether CARP-1 threonine^667 ^regulates apoptosis signaling was investigated next. Cells were either untransduced or transduced with viruses encoding vector, myc-His-CARP-1 (651-759), or myc-His-CARP-1 (651-759^T667A^) proteins for 48 h followed by apoptosis determination as in methods. The cells transduced with myc-His-CARP-1 (651-759) displayed significantly enhanced apoptosis when compared to the apoptosis noted for their vector-transduced or untransduced counterparts (Figure 5C). Expression of myc-His-CARP-1 (651-759^T667A^) protein, on the other hand, resulted in lower levels of apoptosis when compared to apoptosis noted in myc-His-CARP-1 (651-759) transduced cells (Figure 5C). These findings suggest that expression of myc-His-CARP-1 (651-759) protein, like wild-type or CARP-1 (603-898) mutant [4] promotes apoptosis while T667A substitution interferes with this function.

Since H89 treatments caused elevated CARP-1 expression and apoptosis, while CARP-1 interacts with TAZ, and TAZ regulates CARP-1-dependent apoptosis (figures 2, 3), we wished to determine whether and to what extent TAZ regulates H89-dependent apoptosis. For this experiment, we first transduced the HBC cells with viruses encoding vector or mouse TAZ (wt) protein for a period of 12 h. The cells were then either untreated or treated with H89 for additional 48 h, followed by determination of apoptosis. Although, exposure to H89 caused statistically significant increase in apoptosis of vector transduced cells, it failed to promote apoptosis in TAZ-transduced cells (Figure 5D). These findings suggest that TAZ interferes with apoptosis signaling by H89, and together with the data in figures 2, 3 and 4 indicate that CARP-1 threonine phosphorylation following H89 treatment promotes TAZ sequestration by CARP-1 that, in part, contributes to CARP-1-dependent growth suppression by H89.

### Apoptosis signaling by H89 or CARP-1 target c-myc expression

**Figure 6:**
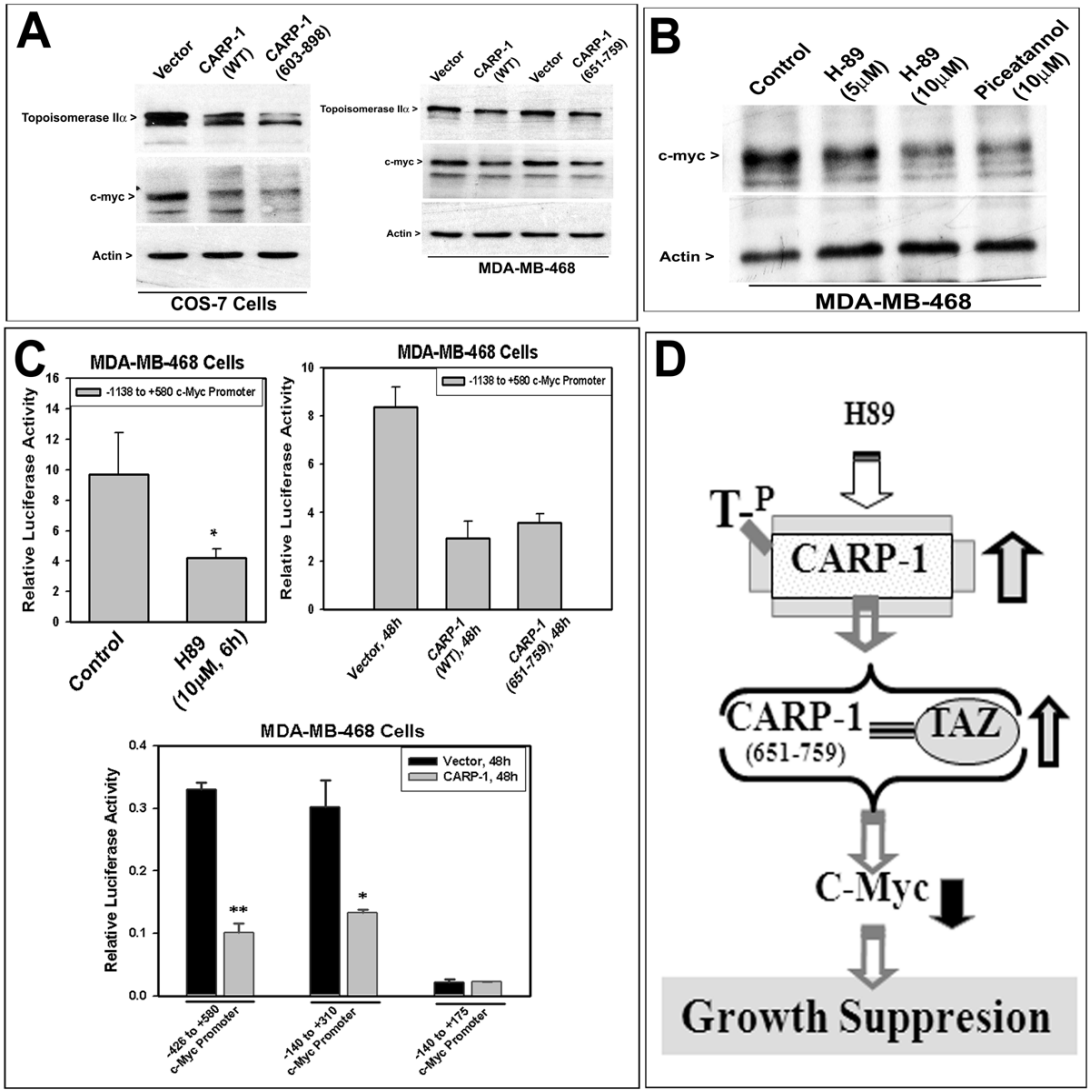
**Expression of CARP-1 (A) or H89 treatment (B) causes reduced c-myc levels, in part by suppressing c-myc transcription (C)**. Cells were transduced with retroviruses encoding vector or CARP-1 proteins as indicated in panel A. For panel B, HBC cells were either untreated or treated with indicated doses of H89 or piceatannol. Approximately 100 μg of respective protein lysate was analyzed by SDS-PAGE, followed by western immunoblotting with either anti c-myc or topoisomerase IIα antibodies essentially as in methods. The membrane was subsequently probed with anti-actin antibody to assess loading. Presence of c-myc, topoisomerase IIα and actin proteins is denoted on the left side of each panel. In panel C, HBC cells were transfected with indicated c-myc promoter-luciferase reporter plasmid. The cells were either untreated (Control) or treated with H89. In addition, the HBC cells were separately transfected with luciferase reporter plasmids having full length c-myc promoter or its various deletions, followed by their transduction with retroviruses encoding vector or CARP-1 protein. The cell lysates were analyzed for luciferase reporter activities as in methods. Columns in the histograms represent means of two independent experiments; bars, SE. Data were analyzed using a two-tailed Student *t *test. For top left histogram, *P < 0.044, compared to the corresponding control, and for the lower histogram, *P < 0.0055 and **P < 0.006, compared to the respective vector-transduced controls. Panel D, Schematic of CARP-1-dependent growth suppression signaling in the presence of H89. = , Binding.

Since we previously found that CARP-1 negatively regulates c-myc expression [3], we wished to determine whether and to what extent expression of cell proliferation regulatory genes such as c-myc and topoisomerase IIα are a target of CARP-1-mediated H89 signaling and the mechanisms involved therein. To test this possibility, we first determined whether expression of c-myc and topoisomerase IIα is inhibited by CARP-1 and its TAZ-interacting mutants. Expression of wild-type CARP-1 or its TAZ-interacting mutants {CARP-1 (603-898) or CARP-1 (651-759) proteins} resulted in reduced levels of c-myc as well as topoisomerase IIα when compared with their vector-transduced counterparts (Figure 6A). Furthermore, treatments with H89 or piceatannol also resulted in diminished c-myc expression in HBC cells when compared with their untreated counterparts (Figure 6B). Since, H89 causes elevated expression of CARP-1 and apoptosis, the findings in figure 6A and 6B suggest that cell growth inhibition following H89 treatments are due, in part, to CARP-1-dependent loss of cell growth promoting factors such as c-myc.

In the next set of experiments, we investigated the mechanism(s) of H89-dependent loss of c-myc expression. Since TAZ is a transcriptional co-activator and CARP-1 is also a regulator of nuclear receptor complex [6,8], we first examined whether CARP-1 induction following H89 treatment attenuates c-myc transcription. To this end, we utilized a plasmid where luciferase reporter is driven by a mouse c-myc promoter from positions -1138 to +580 relative to the P1 transcription start site [18,19]. As expected, significant loss of c-myc promoter activity was noted in H89-treated HBC cells (Figure 6C, top left). The extent of CARP-1 attenuated c-myc transcription was further investigated by transducing c-myc promoter-luciferase reporter-transfected HBC cells with retroviruses encoding vector, wild-type CARP-1, or CARP-1 (651-759) proteins. Expression of wild-type CARP-1 or its TAZ-interacting 651-759 mutant caused significant loss of c-myc promoter activity when compared with their vector-transduced counterparts (Figure 6C, top right). Additional transfection studies revealed that CARP-1/TAZ-responsive element(s) is located within -140 to +310 region of the mouse c-myc promoter (Figure 6C, lower). Taken together, data in figure 6 demonstrate that attenuation of c-myc expression by H89 or CARP-1 is accomplished, in part, by transcriptional down-regulation of c-myc, and contributes to the growth suppressing effects of CARP-1 and H89.

## Discussion

We previously reported identification and characterization of CARP-1/CCAR1, a peri-nuclear phospho-protein that mediates apoptosis signaling by diverse agents including adriamycin and inhibitors of EGFRs [3,4]. The micro-array-based approach revealed that CARP-1-dependent cell growth inhibition involves cell cycle regulatory proteins such as c-Myc and cyclin B1 [3]. Since attenuation of PKA often inhibits cell growth we wished to investigate whether CARP-1 was involved in this signaling. Our studies revealed that H89, a commonly utilized pharmacologic inhibitor of PKA-Cα, inhibited cell growth in part by inducing apoptosis. The fact that a number of recent studies have implicated H89 as an inhibitor of other kinases such as the MSK1 and the ribosomal S6Kinase [reviewed in ref [14]], we first clarified whether apoptosis induction by H89 involved its targeting of PKA-Cα, MSK1, or S6Kinases. Surprisingly, Si-RNA-dependent knock-down of either of these kinases failed to induce apoptosis in HBC cells. Furthermore, HBC cells that were treated with various doses of R_p_-cAMPS, a competitive antagonist of the cyclic nucleotide-binding domain on PKA, over a range of time periods also failed to stimulate apoptosis (not shown). Our Si-RNA-based experiments therefore suggest that H89 likely inhibits cell growth independent of its targeting of the PKA-Cα, MSK1, or S6Kinases and may point to additional cell growth inhibitory mechanisms utilized by this agent. The fact that CARP-1 was involved in transducing H89 signaling as noted in our current studies, we propose that CARP-1-dependent H89 inhibition of HBC cell growth is a novel pathway (Figure 6D).

CARP-1 and its mutants suppress cell growth in part by activating intrinsic apoptosis pathway [4]. Moreover, CARP-1 interacted with cytoplasmic signaling transducer 14-3-3/Stratifin protein, while over-expression of stratifin interfered with CARP-1-dependent apoptosis [3]. An array of cell growth regulators such as Raf, PI-3 kinase, MEKK, BAD, ASK-1, cdc25, CDK25, p53, FKHRL, and TAZ serve as substrates for binding with 14-3-3 proteins. TAZ, in particular, interacts with 14-3-3 protein in a phsophorylation-dependent manner, and in the light of our data demonstrating CARP-1 binding with TAZ, and the fact that overexpression of TAZ attenuates CARP-1-dependent apoptosis (figures 4 and 5), suggests for existence of a dynamic multiprotein cytoplasmic complex that functions to regulate cell growth. This complex is a target of phosphorylation signaling by H89 is further supported by our data in figure 5 showing TAZ-binding sub-domain of CARP-1 (CARP-1 651-759) is a target of threonine phopshorylation by H89, and that the threonine^667 ^regulates CARP-1 binding with TAZ and subsequent apoptosis.

Since, CARP-1 has a novel serine-phosphorylation motif [16], and the fact that 14-3-3 proteins often bind to serine/threonine-phosphorylated residues in their target proteins [20,21] it is unclear whether CARP-1 interactions with 14-3-3/Stratifin are dependent on prior specific serine/threonine phosphorylation of CARP-1. CARP-1 binding with TAZ, on the other hand, is dependent on phosphorylation of CARP-1 threonine^667 ^without the involvement of TAZ WW, PDZ domain protein binding, and 14-3-3-interacting motifs as reflected by our data in figure 4. Since TAZ appears to have a novel motif for binding with CARP-1, it is likely that TAZ mediates CARP-1 sequestration by 14-3-3 in a manner dependent on phosphorylation of both the TAZ and CARP-1 proteins, in turn promoting the formation of a multiprotein cytoplasmic complex. The enhanced threonine phosphorylation and expression of CARP-1 in the presence of H89 likely favors cytoplsmic sequestration of TAZ, and consequently interferes with nuclear translocation and transcriptional co-activation function of TAZ.

A range of transcription factors target specific cis-sequences within the c-myc promoter and enhancer region in a manner that is often dependent on the cell type as well as context of the signaling pathway [22]. For example, PDGF-dependent mitogenic signaling regulates c-Myc expression through multiple pathways that target c-myc promoter elements [23,24]. The PDGF activates c-myc transcription through a JNK and AP-1/c-fos/c-Jun-dependent pathway that targets a canonical AP-1 consensus element located ~1.3 kb upstream of the P1 start site [23], while PDGF-dependent c-myc expression is also regulated through Rho GTPases involving a region from -157 to +500 relative to P1 transcription start site of c-myc promoter [24]. In the latter case, however, the precise nature of the cis-trans elements remains to be elucidated. Although, TAZ is a transcriptional co-activator of proteins with PPXY motifs, that include a growing list of transcription factors such as c-Jun [8], and the fact that CARP-1-TAZ interactions following H89 treatments interfere with c-myc transcription (figure 6) while the AP-1 consensus sequences are located outside of the region targeted by H89 or CARP-1 (see figure 6) suggest that H89-dependent TAZ-CARP-1 signaling is unlikely to interfere with AP-1 function. However, of note is the fact that the H89-responsive mouse c-myc promoter region (-140 to +310 relative to P1 start site, figure 6) overlaps with -157 to +500 region of human c-myc promoter sequences that are targeted by PDGF through the Rho GTPases. Whether the two signaling pathways indeed share a common transcription factor(s) that target known and/or novel cis sequences remains to be clarified.

## Conclusion

H89 utilizes a novel pathway to regulate HBC cell growth in a manner involving elevated threonine phosphorylation of CARP-1, its binding with a transcriptional co-activator TAZ, and repression of c-myc.

## Methods

### Materials

DMEM, Ham's F-12 medium, fetal bovine serum (FBS), and lipofectamine-based transfection kit were purchased from Life Technologies, Inc., Grand Island, NY. Piceatannol, H89, R_p_-cAMPS (a competitive antagonist of the cyclic nucleotide-binding domain on PKA), and S_p_-cAMPS (a competitive agonist of the cyclic nucleotide-binding domain on PKA) were obtained from Calbiochem/EMD Biosciences (San Diego, CA). The anti-PKA Cα, anti-phospho-PKA C (Thr197) polyclonal antibodies, anti-MSK1, anti-ribosomal S-6-kinase, as well as anti-myc-tag mouse monoclonal (9B11) antibodies were purchased from Cell Signaling (Beverley, MA). Anti-flag M2 and anti-c-myc (clone 67P05) monoclonal antibodies were obtained from Sigma-Aldrich, (St. Louis, MO) and Lab Vision (Fremont, CA), respectively. Anti-phospho-tyrosine and anti-phospho-serine antibodies were purchased from Zymed labs (San Francisco, CA), while Anti-phospho-threonine (H-2) HRP conjugate were from Santa Cruz Biotech (Santa Cruz, CA). Anti-topoisomerase IIα mouse monoclonal (3F6) antibody was from Novacastra/Vector laboratories, Burlingame, CA, and anti-Gst rabbit polyclonal antibody was purchased from Upstate Biotech, Lake placid, NY. Phospho-enrichment beads were purchased from BD Biosciences (Palo Alto, CA).

### Recombinant plasmid constructs

The construction of plasmid clone 6.1 expressing myc-His-tagged wild-type CARP-1 as well as clone 1.6 expressing CARP-1 antisense has been described before [3]. The plasmids pEF-TAZ-N-Flag, pEF-TAZ(-4)-N-Flag, and pEF-TAZ(S89A)-N-Flag that encode wild type or mutant TAZ proteins were as detailed before [8]. The plasmids encoding N-terminal gst epitope tagged wild type or mutants TAZ proteins were generated by PCR amplification using mouse TAZ cDNA as template followed by their cloning into the pEBG vector plasmid. The plasmid pEBG-TAZ (wt) encodes Gst-tagged wild-type TAZ, while pEBG-TAZ (1-164), pEBG-TAZ (165-395), and pEBG-TAZ (1-120) encode Gst-tagged truncated mouse TAZ proteins. Of note is that fact that the mutant TAZ (1-120) lacks the WW domain that has been implicated in TAZ interactions with PPXY motif of nuclear transcription factors such as c-Jun [8]. In addition, recombinant plasmids for expression of mutant CARP-1 proteins were generated by a combination of standard PCR amplification and cloning methodologies to generate constructs that encode CARP-1 proteins having myc and His tags positioned at the carboxyl termini. Constructs encoding CARP-1 (651-759) protein having threonine at position 667 substituted with alanine or aspartate were similarly generated using a combination of PCR amplification and cloning methods. All the recombinant plasmids were sequenced to ascertain validity of the cDNA inserts. The plasmids having mouse c-myc promoter (-1138 to +580, -426 to +580, -140 to +310, and -140 to +175 relative to the P1 transcription site) driving luciferase reporter gene have been described before [18,19].

### Cell Lines and Cell Culture

ER-negative, p53-negative SKBR-3, MDA-MB-468 HBC cells, and their sublines expressing CARP-1 antisense (Clone 1.6 sublines 1, 4, 9, and 10), as well as human colon cancer HCT-116 cells were cultured and maintained as detailed before [3,19]. HCT-116 sublines stably expressing vector plasmid or myc-His-tagged wild type CARP-1 were isolated and characterized as described before [4]. Monkey kidney COS-7 and NIH3T3 mouse fibroblast cells were obtained from ATCC (Manassas, VA) and propagated per vendor's suggestions. NIH3T3 derivative PT-67 mouse fibroblasts were purchased from BD-Clontech (Palo Alto, CA), and generation and characterization of their stable isolates expressing vector pLNCX2, myc-His-tagged wild-type or mutant CARP-1 proteins was carried out as previously described [3,4].

### Luciferase assays

Cells were plated either in a 60-mm dish or in a 6-well plate at a density of 3 × 10^5 ^cells/ml and then co-transfected with pTK/Renilla Luc in combination with various c-myc-Promoter-luc constructs essentially following previously detailed methods [3,4,25]. After 6-h incubation with plasmid DNAs, cells were washed three times with 1 × PBS, co-incubated with retroviruses encoding vector, wild-type or mutant CARP-1 proteins, and allowed to grow in the appropriate culture media for additional 48 h. The cells were then harvested, lysed, and Renilla and firefly luciferase activities were measured using dual luciferase assay kit (Clontech) essentially following vendors guidelines.

### Gene Knock-down experiments

CARP-1 expression was down-regulated in HBC cells by stably expressing a plasmid expressing CARP-1 antisense cDNA. Antisense transfected sublines 1, 4 express normal levels, while sublines 9 and 10 express 50% reduced levels of CARP-1 and their characterization has been detailed before [3]. Depletion of PKA-Cα, CARP-1, S6K, or MSK1 was carried out by transfecting MDA-MB-468 HBC cells with scrambled (non-target) or respective specific on-target Si-RNAs (Dharmacon) following manufacturer suggested guidelines. Seventy-two hours post-transfections, the cell lysates were analyzed by western immunoblotting in conjunction with respective antibodies essentially following the methodologies detailed below.

### Immunoprecipitation, Western Blot, Apoptosis, and Co-localization Assays

Logarithmically growing cells were either untransfected or transfected with various plasmids for 24 or 48 hours followed by their lysis to prepare protein extracts. In certain instances cells were treated with H89. For immunoprecipitation aliquots of cell lysates containing 1.0 mg proteins were incubated with indicated antibodies and Sepharose G at 4°C for 3 hours. Immunoprecipitates or the protein extracts were then analyzed by western blotting using indicated antibodies per the manufacturer's guidelines and previously published protocols [3,4,19]. For enrichment of phosphorylated proteins, the cells were first transfected with plasmids encoding wild-type or mutant CARP-1 proteins, followed by treatments of cells with H89. Equal amounts (1 mg) of cell lysates were then applied to phospho-protein enrichment columns; the complexes were washed and eluted following manufacturer suggested methodology. The enriched phospho-proteins alongwith the protein lysate from transfected cells were analyzed by western blotting first with anti-myc tag antibodies followed by reprobing with anti-phospho-tyrosine antibodies. In addition, cell lysates were prepared from untreated as well as treated cells to determine apoptosis by utilizing cell death detection ELISA kit (Roche Diagnostics). The net absorbances at the suggested wavelengths (A_405_nm minus A_495_nm) were obtained for the lysates derived from treated as well as untreated cells. The histograms indicating levels of apoptosis were generated either by plotting net absorbances or by calculating the "enrichment factor" as described [3,4]. In certain instances, cell death was measured by counting acridine orange-ethidium bromide stained cells [25], while viabilities of untreated and treated cells were also measured by utilizing LIVE/DEAD viability/cytotoxicity assay (Molecular Probes, Eugene, OR).

COS-7 cells were transfected with plasmid expressing myc-His-tagged wild-type CARP-1 (CARP-1 clone 6.1; ref. [3]) in combination with plasmid expressing wild-type TAZ (pEF/mTAZ-N-Flag, ref. [8]). The cells were trypsinized, fixed, and permeablizied followed by incubation with a combination of anti-Flag tag mouse monoclonal and anti-myc tag rabbit polyclonal antibodies at 4°C for overnight. The cells were washed again in 1 × PBS for three times, and incubated with a combination of rhodamine conjugated goat-anti-rabbit and Alexa 488 conjugated goat-anti-mouse IGg for a period of 30 min at 37°C. The cells were washed twice with pre-warmed 1 × PBS, and coverslips were mounted with an aqueous-based anti-fade medium. The cell fluorescence was detected by an Olympus BX51 fluorescent microscope fitted with a triple DAPI/FITC/TRITC cube, and the images were captured by a DP70 digital camera and image acquisition software. The exposure time and magnification (400×) were maintained throughout the capture of images. The images were subsequently analyzed by Image analysis and Adobe Photoshop software.

## Abbreviations

CARP-1/CCAR1: Cell cycle and apoptosis regulatory protein 1/Cell division cycle and apoptosis regulator 1; HBC: Human breast cancer; H89: *N*-[2-(*p*-bromocinnamylamino) ethyl]-5-isoquinolinesulfonamide; TAZ: Transcriptional co-activator of PDZ binding proteins; ER: estrogen receptor; GR: glucocorticoid receptor; PKA: Protein kinase A; R_p_-cAMPS: R_p_-adenosine-3', 5'-cyclic monophosphorothioate; S_p_-cAMPS: S_p_-adenosine-3', 5'-cyclic monophosphorothioate; PCR: Polymerase chain reaction; EGFR: epidermal growth factor receptor; S6K: ribosomal S6 kinase; MSK: mitogen and stress-activated kinase.

## Competing interests

The authors declare that they have no competing interests.

## Authors' contributions

YJ carried out majority of experiments including western blots, immunoprecipitations, apoptosis assays, transfections and luciferase assays. VTP and WW carried out SiRNA and some western blot experiments, respectively while LZ carried out CARP-1/TAZ mapping experiments to determine their interacting epitopes. MBY provided various mutants of TAZ, and MBY, AW and JAF participated in design and discussion of certain experiments. AKR subcloned various CARP-1 mutants, and is the corresponding author responsible for experimental design and coordination, as well as writing of the manuscript and formatting of all the figures. All authors read and approved the final manuscript.

## Supplementary Material

Additional file 1 **Supplemental Figure**. CARP-1 (651-759) binds with TAZ (1-120). Cells were transfected with plasmid encoding flag-tagged wild-type TAZ in combination vector or plasmids expressing noted myc-His-tagged CARP-1 mutant proteins (panel A), or the indicated combinations of myc-His-tagged CARP-1 (651-759) and gst-tagged TAZ mutants (panel B). The immunoprecipitation and western blotting were carried out using noted antibodies essentially as in figure 3. In panel A, the membranes were subsequently probed with anti-myc-tag antibodies to assess expression of respective CARP-1 mutant protein. Click here for file

## References

[B1] Zong W-X Thompson CB Necrotic death as a cell fate Genes & Develop 2006 20 1 15 10.1101/gad.1376506 16391229

[B2] Nelson DA White E Exploiting different ways to die Genes & Develop 2004 18 1223 1226 10.1101/gad.1212404 15175258

[B3] Rishi AK Zhang L Boyanapalli M Wali A Mohammad RM Yu Y Fontana JA Hatfield JS Dawson MI Majumdar APN Reichert U Identification and characterization of a cell cycle and apoptosis regulatory protein-1 as a novel mediator of apoptosis signaling by retinoid CD437 J Biol Chem 2003 278 33422 33435 10.1074/jbc.M303173200 12816952

[B4] Rishi AK Zhang L Yu Y Jiang Y Nautiyal J Wali A Fontana JA Levi E Majumdar APN Cell cycle- and apoptosis-regulatory protein-1 is involved in apoptosis signaling by epidermal growth factor receptor J Biol Chem 2006 281 13188 13198 10.1074/jbc.M512279200 16543231

[B5] Matsuoka S Ballif BA Smogorzewska A McDonald ER III Hurov K Luo J Bakalarski CE Zhao Z Solimini N Lerenthal Y Shiloh Y Gygi SP Elledge SJ ATM and ATR substrate analysis reveals extensive protein networks responsive to DNA damage Science 2007 316 1160 1166 10.1126/science.1140321 17525332

[B6] Kim JH Yang CK Heo K Roeder RG An W Stallcup MR CCAR1, a key regulator of mediator complex recruitment to nuclear receptor transcription complexes Molecular Cell 2008 31 510 519 10.1016/j.molcel.2008.08.001 18722177 PMC2562329

[B7] Tzivion G Shen YH Zhu J 14-3-3 proteins; bringing new definitions to scaffolding Oncogene 2001 20 6331 6338 10.1038/sj.onc.1204777 11607836

[B8] Kanai F Marignani PA Sarbassova D Yagi R Hall RA Donowitz M Hisaminato A Fujiwara T Ito Y Cantley LC Yaffe MB TAZ: a novel transcriptional co-activator regulated by interactions with 14-3-3 and PDZ domain proteins The EMBO J 2000 24 6798 6791 10.1093/emboj/19.24.6778 PMC305881 11118213

[B9] Hong JH Hwang ES McManus MT Amsterdam A Tian Y Kalmukova R Mueller E Benjamin T Spiegelman BM Sharp PA Hopkins N Yaffe MB TAZ, a Transcriptional Modulator of Mesenchymal Stem Cell Differentiation Science 2005 309 1074 1078 10.1126/science.1110955 16099986

[B10] Lei QY Zhang H Zhao B Zha ZY Bai F Pei XH Zhao S Xiong Y Guan KL TAZ Promotes Cell Proliferation and Epithelial-Mesenchymal Transition and Is Inhibited by the Hippo Pathway Mol Cell Biol 2008 28 2426 2436 10.1128/MCB.01874-07 18227151 PMC2268418

[B11] Chin K-V Yang W-L Ravtin R Kita T Reitman E Vettori D Cvijic ME Shin M Iacono L Reinventing the wheel of cyclic AMP: Novel mechanisms of cAMP signaling Ann NY Acad Sci 2002 968 49 64 10.1111/j.1749-6632.2002.tb04326.x 12119267

[B12] Engh RA Girod A Kinzel V Huber R Bossemeyer D Crystal structures of catalytic subunit of cAMP-dependent protein kinase in complex with isoquinolinesulfonyl protein kinase inhibitors H7, H8, and H89. Structural implications for selectivity J Biol Chem 1996 271 26157 26164 10.1074/jbc.271.42.26157 8824261

[B13] Taylor SS Buechler JA Yonemoto W cAMP-dependent protein kinase: framework for a diverse family of regulatory enzymes Annu Rev Biochem 1990 59 971 1005 10.1146/annurev.bi.59.070190.004543 2165385

[B14] Murray AJ Pharmacological PKA inhibition: All may not be what it seems Sci Signal 2008 1 Re4 10.1126/scisignal.122re4 18523239

[B15] Tinnikov AA Yeung KT Das S Samuels HH Identification of a novel pathway that selectively modulates apoptosis of breast cancer cells Cancer Res 2009 69 1375 1382 10.1158/0008-5472.CAN-08-2896 19190336 PMC4264605

[B16] Beausoleil SA Jedrychowski M Schwartz D Elias JE Villen J Li J Cohn MA Cantley LC Gygi SP Large-scale characterization of HeLa cell nuclear phosphoproteins Proc Natl Acad Sci 2004 101 USA 12130 12135 10.1073/pnas.0404720101 15302935 PMC514446

[B17] Combet C Blanchet C Geourjon C Deleage G NPS@: network protein sequence analysis Trends Biochem Sci 2000 25 3 147 150 10.1016/S0968-0004(99)01540-6 10694887

[B18] Ramana CV Grammatikakis N Chernov M Nguyen H Goh KC Williams BRG Stark GR Regulation of c-myc expression by IFN-( through Stat1-dependent and -independent pathways The EMBO Journal 2000 19 263 272 10.1093/emboj/19.2.263 10637230 PMC305560

[B19] Zhang L Wali A Ramana CV Rishi AK Cell growth inhibition by Okadaic acid involves Gut-enriched Kruppel-like factor-mediated enhanced expression of c-Myc Cancer Research 2007 67 21 10198 206 10.1158/0008-5472.CAN-07-2505 17974960

[B20] Zha J Harada H Yang E Jockel J Korsmeyer SJ Serine phosphorylation of death agonist BAD in response to survival factor results in binding to 14-3-3 not BCL-X(L) Cell 1996 87 619 628 10.1016/S0092-8674(00)81382-3 8929531

[B21] Datta SR Dudek H Tao X Masters S Fu H Gotoh Y Greenberg ME Akt phosphorylation of BAD couples survival signals to the cell-intrinsic death machinery Cell 1997 91 2 231 41 10.1016/S0092-8674(00)80405-5 9346240

[B22] Marcu KB Bossone SA Patel AJ myc function and regulation Annu Rev Biochem 1992 61 809 860 10.1146/annurev.bi.61.070192.004113 1497324

[B23] Iavarone C Catania A Marinissen MJ Visconti R Acunzo M Tarantino C Carlomagno MS Bruni CB Gutkind JS Chiariello M The Platelet-derived Growth Factor Controls c-*myc *Expression through a JNK- and AP-1-dependent Signaling Pathway J Biol Chem 2003 278 50024 50030 10.1074/jbc.M308617200 14523011

[B24] Chiariello M Marinissen MJ Gutkind JS Regulation of c-myc expression by PDGF through Rho GTPases Nature Cell Biol 2001 3 580 586 10.1038/35078555 11389443

[B25] Ausubel FM Brent R Kingston RE Moore DD Seidman JG Smith JG Struhl K Eds Current protocols in molecular biology 1990 2

