# Supplementary figures and images for: A novel mechanism of cell growth regulation by Cell Cycle and Apoptosis Regulatory Protein (CARP)-1
Source: J Mol Signal. 2010 Jul 1;5:7. doi: 10.1186/1750-2187-5-7 (PMC2904743; doi:10.1186/1750-2187-5-7)

## Slide 1
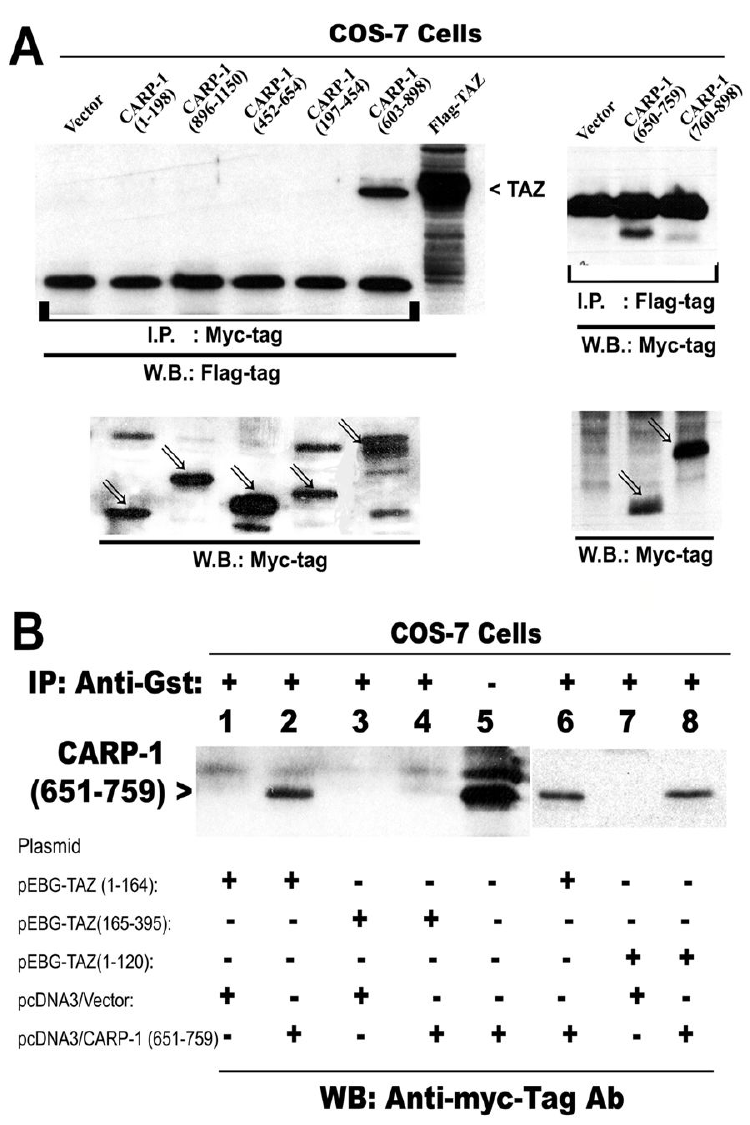

Supplement: Additional file 1 — Supplemental Figure. CARP-1 (651-759) binds with TAZ (1-120). Cells were transfected with plasmid encoding flag-tagged wild-type TAZ in combination vector or plasmids expressing noted myc-His-tagged CARP-1 mutant proteins (panel A), or the indicated combinations of myc-His-tagged CARP-1 (651-759) and gst-tagged TAZ mutants (panel B). The immunoprecipitation and western blotting were carried out using noted antibodies essentially as in figure 3. In panel A, the membranes were subsequently probed with anti-myc-tag antibodies to assess expression of respective CARP-1 mutant protein. [file 1750-2187-5-7-S1.PPT]
